# Pediatric Extensor Pollicis Longus Rupture After Distal Radius Fracture: A Case Report

**DOI:** 10.7759/cureus.113165

**Published:** 2026-07-22

**Authors:** Carson B Twiss, Max Jiganti, Robert Umberhandt, Shelby Mills

**Affiliations:** 1 Orthopedic Surgery, Good Samaritan Regional Medical Center, Corvallis, USA; 2 Pediatric Orthopedics, Mary Bridge Children's Hospital, Tacoma, USA; 3 Orthopedic Surgery, Western University of Health Sciences, Lebanon, USA

**Keywords:** extensor pollicus longus rupture, pediatric distal radius fracture, pediatric orthopedic surgery, salter harris type 2, tendon transfer

## Abstract

Extensor pollicis longus (EPL) tendon rupture is a significant complication following a distal radius fracture that is well reported in the adult patient population but rarely seen in the pediatric patient population. We present a delayed EPL tendon rupture in a skeletally immature patient with a minimally displaced distal radius fracture and aim to highlight the importance of prompt diagnosis and appropriate management of this uncommon complication. We report a case of a 14-year-old female who presented with a Salter-Harris type II fracture of the distal radius with 10 degrees of dorsal angulation. Following non-operative treatment with brace immobilization, the patient presented at four weeks with an atraumatic rupture of her EPL tendon. Subsequently, she underwent extensor indicis proprius (EIP) transfer. She underwent follow-up at two months, five months, and one year. Only two other cases of a delayed EPL rupture following a minimally displaced distal radius fracture in a skeletally immature patient managed non-operatively have been reported. This case illustrates a successful outcome in a pediatric patient managed with an EIP tendon transfer.

## Introduction

Distal radius fractures are one of the most common musculoskeletal injuries, and the most common fracture type in the pediatric population [1]. Pediatric skeletal immaturity allows for significant bone remodeling to occur, and many of these fractures can be treated conservatively when operative criteria are not met. There is no clear consensus regarding operative criteria; however, some guidelines have suggested tolerances for non-operative management with up to 20 degrees of dorsal-volar angulation for kids older than 10 [2]. A rare complication of distal radius fracture includes extensor pollicis longus (EPL) rupture, reported in up to 5% of cases in adults [3-5]; however, this complication is rarely seen in the pediatric population [6,7]. A 2020 literature review found only one case of an EPL rupture in a minimally displaced distal radius fracture in a skeletally immature patient managed non-operatively [8]. A proposed mechanism of tendon rupture is due to attritional tears as the EPL passes over callus from healing fractures or from hardware that is prominent if surgical fixation is used. Risk factors for attritional EPL rupture in distal radius fractures include inflammatory conditions (e.g., rheumatoid arthritis), corticosteroid use, or surgical fixation with plating [4,9]. EPL rupture causes loss of extension of the thumb at the interphalangeal (IP) joint that, without surgical intervention, is not otherwise restored.

There are multiple methods of treatment for EPL rupture, including direct repair, tendon grafting, or tendon transfer. This study focuses on management of an atraumatic EPL rupture with tendon transfer of extensor indicis proprius (EIP) [10,11]. EIP tendon transfers have been shown to have high rates of patient satisfaction, and while there have been reports of patients with residual loss of strength with extension of the index finger following EIP transfer, this does not cause disruption to daily activities [12-15].

Few reports exist involving pediatric patients with delayed rupture of EPL following a distal radius fracture. Most studies involved either a significantly displaced fracture pattern or management with surgical fixation. Brooker et al. report a case series of nine pediatric patients who sustained EPL ruptures following dorsal entry flexible nailing of radial shaft fractures [16]. A study by Patel et al. reports a skeletally immature patient with an isolated EPL rupture following surgical fixation of a Salter-Harris II distal radius fracture [17]. To our knowledge, only two studies by Song et al. and Munaretto et al. involve a delayed EPL rupture following a minimally displaced, non-operatively managed Salter-Harris II fracture, though overall similar reports remain limited [8,18,19].

This case report of a pediatric patient with an EPL rupture after a conservatively treated Salter Harris II distal radius fracture aims to add to the paucity of literature surrounding this complication.

## Case presentation

**Figure 1:**
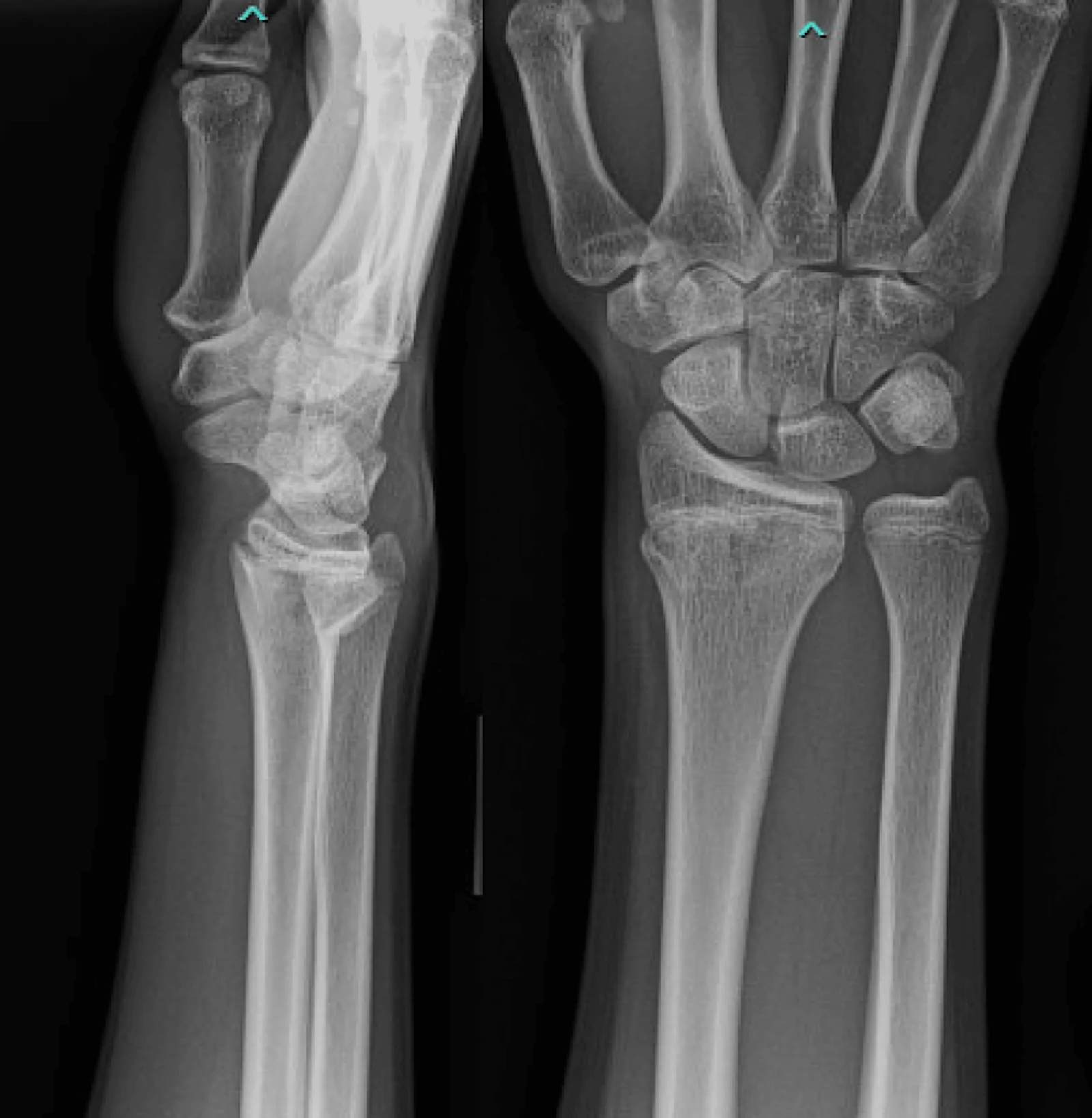
Initial injury films, anteroposterior (AP) and lateral views of the right wrist.

**Figure 2:**
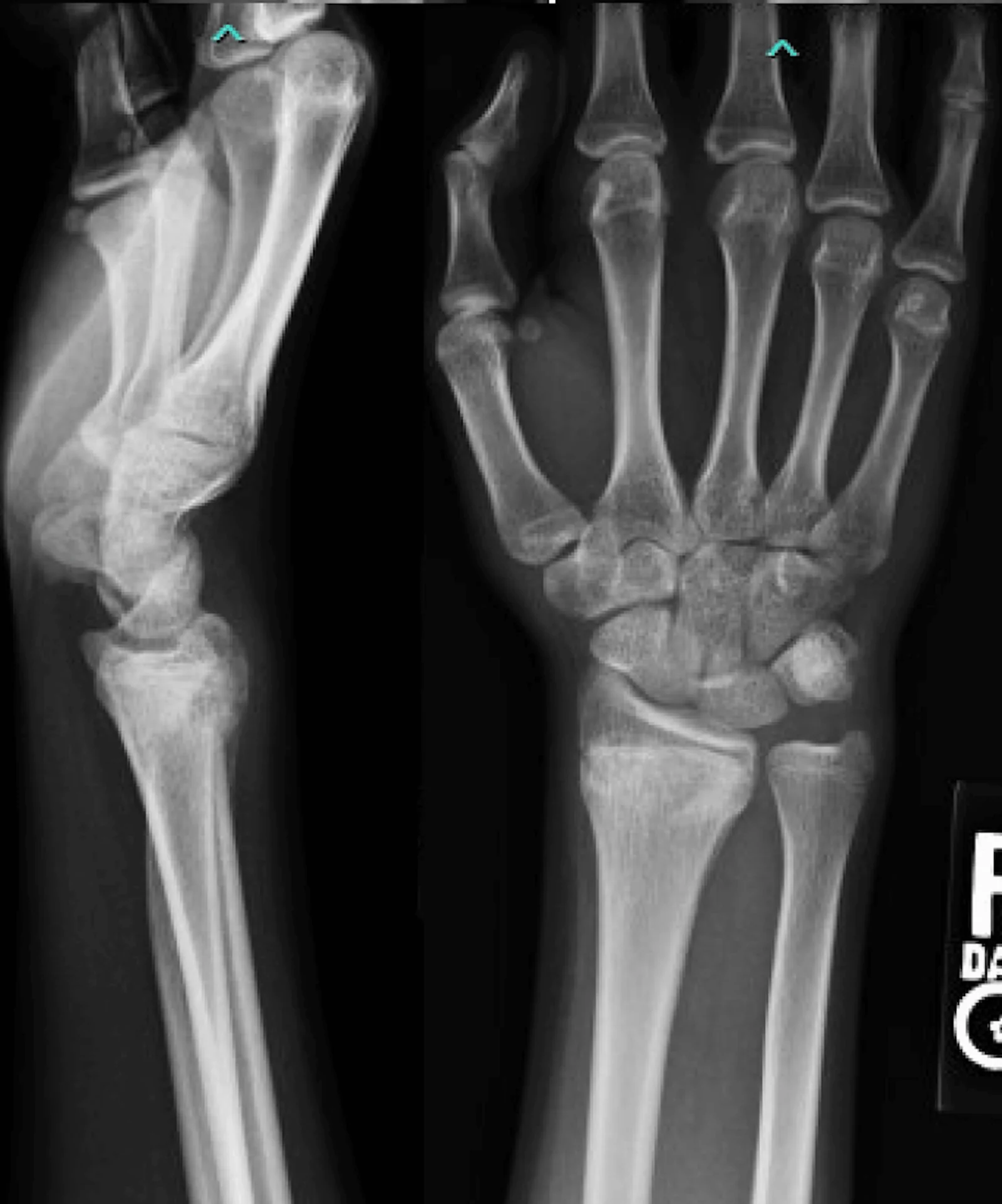
Plain films obtained in clinic at time of evaluation, when EPL rupture was diagnosed. EPL, extensor pollicis longus

A 14-year-old female presented to the emergency department after falling off a horse and landing on her right arm. X-rays confirmed a Salter-Harris type II fracture of the distal radius with 10 degrees of dorsal angulation (Figure 1), and the patient was reduced and splinted in the emergency department. The splint was then exchanged for a removable wrist brace at her first follow-up appointment. Four weeks later, the patient experienced a pop in her wrist when playing in the yard. She presented to the clinic several days later with the inability to extend the interphalangeal joint of her right thumb. X-rays were taken at that visit and revealed a healed fracture with dorsal callus formation (Figure 2). The patient was diagnosed with an EPL tendon rupture, and the decision was made to proceed with surgical management for an EIP tendon transfer.

**Figure 3:**
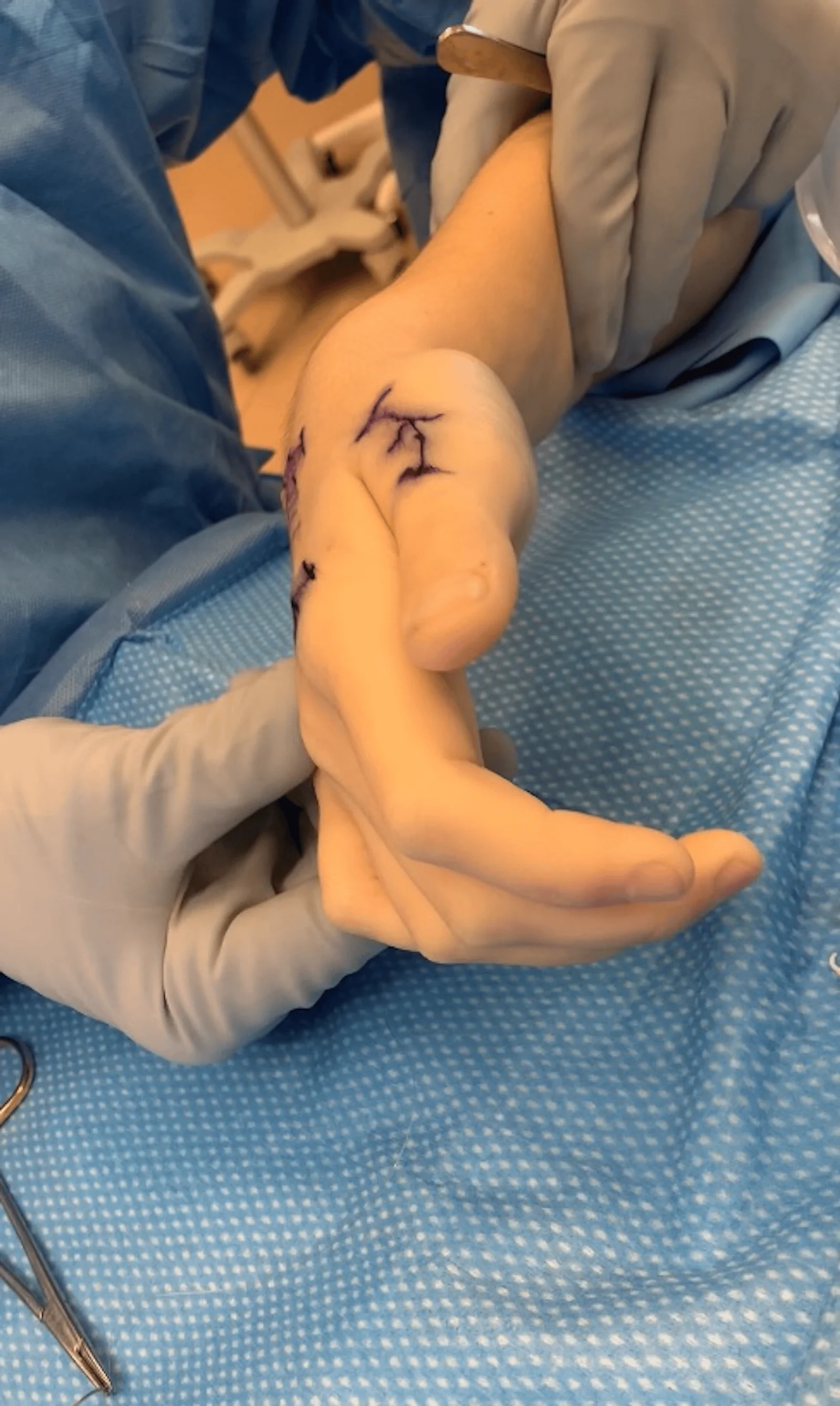
Intraoperative photo demonstrating appropriate tenodesis effect, with extension of the thumb interphalangeal (IP) joint during passive wrist flexion.

In the operative suite, a linear vertical incision over Lister’s tubercle was made, and the EPL was found to be frayed proximally and adherent to surrounding tissue, with the distal stump just distal to Lister’s tubercle. Dorsal incisions were made to identify and transect the EIP. With the wrist extended approximately 30 degrees and the thumb fully extended, the EIP was approximated to the distal aspect of the proximal EPL tendon limb using a Pulvertaft weave secured with 4-0 non-absorbable braided suture. The tenodesis exam was performed, confirming thumb IP joint extension with wrist flexion and thumb IP joint flexion with wrist extension (Figure 3). The patient was placed in a thumb spica cast.

The patient followed up in clinic for cast removal at four weeks and was transitioned to a removable Velcro brace, with instructions to continue bracing for an additional four weeks. Occupational therapy was started, and at two months, she noted significant subjective improvements as well as full thumb IP joint flexion and extension. Immobilization was discontinued at this time. At five-month follow-up after the tendon transfer surgery, the patient was using her thumb normally and had returned to her regular daily activities, including playing piano and participating in sports. A final follow-up was conducted via telemedicine conference one year out from surgery, and the patient was overall happy with the results from surgery; however, she reported no longer playing basketball due to wrist discomfort on the operative side.

## Discussion

EPL rupture in a non-operatively managed distal radius fracture in a pediatric patient is a rare complication with few cases reported in the literature. This paper aims to present this rare complication in a patient without typical risk factors, the subsequent operative management, and a report of the outcomes. Given the dorsal callus formation seen both radiographically and intraoperatively, the proposed mechanism of this patient’s EPL rupture was attritional compromise of the integrity of the tendon with repetitive sliding over the dorsal callus. It is possible the removable wrist brace provided to this patient at initial follow-up allowed more motion compared to cast immobilization, contributing to more callus formation and tendon irritation. While there is no literature we are aware of that addresses differences in callus formation between removable bracing or casting of pediatric distal radius fractures, the literature does support non-inferiority of bracing in minimally displaced fractures with less than 10 degrees of angulation [20].

Successful management of this complication relied on prompt recognition and surgical reconstruction. EPL rupture treatment with EIP tendon transfer has proven to be a successful treatment method, with well-reported patient satisfaction scores and return of strength and function following surgery [12-15]. While there are no specific recommendations in the literature regarding timing to surgical correction, successful outcomes have been reported in cases up to 15 months out from the initial injury [21]. Our patient underwent surgery with EIP tendon transfer within two months of the injury and had full return to activity three months after surgery with continued positive results one year out.

## Conclusions

We propose that, while rarely reported in the literature, EPL rupture in conservatively managed distal radius fractures in the pediatric patient is a serious complication that requires surgical intervention to restore extension at the thumb IP joint. Further studies, such as a pediatric registry or multi-center study looking at a larger cohort of patients with this complication, could better identify common risk factors, management options, and outcomes.
